# Diets, Lifestyles and Metabolic Risk Factors among Corporate Information Technology (IT) Employees in South India

**DOI:** 10.3390/nu15153404

**Published:** 2023-07-31

**Authors:** Paromita Banerjee, G. Bhanuprakash Reddy, Hrusikesh Panda, Kiran Kumar Angadi, Thirupathi Reddy, SubbaRao M. Gavaravarapu

**Affiliations:** 1 Nutrition Information, Communication & Health Education (NICHE) Division, ICMR-National Institute of Nutrition, Hyderabad 500007, Telangana, India; paromitabanerjee0806@gmail.com (P.B.); hrusinin@gmail.com (H.P.); thirupathireddy2423@gmail.com (T.R.); 2 Biochemistry Division, ICMR-National Institute of Nutrition, Hyderabad 500007, Telangana, India; geereddy@yahoo.com (G.B.R.); dr.kirankumarangadi@gmail.com (K.K.A.)

**Keywords:** employee health, workplace wellness

## Abstract

(1) Information Technology (IT) Business Process Outsourcing (BPO), the largest employment sector of India, contributes to rapid economic growth. However, the work of IT employees is sedentary, and the food environments of their worksites expose them to an obesogenic environment. This study aimed to assess their metabolic and lifestyle risk factors. (2) Methods: To examine the health and nutrition status of IT employees, anthropometric, biochemical and clinical assessments were conducted among 183 employees from three IT organizations of varied operational sizes. Their health-, diet- and physical activity-related practices were assessed using a questionnaire. The prevalence of MetS was assessed. Selected biomarker levels were assessed and associated with their self-perceived stress levels. (3) Results: The median age of the employees was 30 years (26–35 years). While 44.02% of employees were overweight, 16.85% of employees were obese. About 3.89% of employees were found to be diabetic, and HDL-C levels were lower than recommended in 64.93% of employees. In all, 29.87% of the study population were considered to have metabolic syndrome since they had metabolic risk scores ≥ 3. Those with metabolic syndrome were significantly older (*p* = 0.000), and levels of MDA (*p* = 0.003), homocysteine (*p* = 0.001), IL-6 (*p* = 0.017) and IL-4 (*p* = 0.000) were significantly higher among them. Although the prevalence of MetS was significantly lower among those aged >30 years, the lifestyle risk factors were significantly higher among them. (4) Conclusions: The assessed parameters indicate a high risk of developing NCDs among employees in the IT industry in India. This shows the need for the modification of lifestyle and workplace food and physical activity environments.

## 1. Introduction

The Information Technology (IT) industry has grown exponentially in the last few decades in India [1,2,3], and its tech-savvy workforce has contributed significantly to the country’s socioeconomic development. With over 4.14 million people being employed and 0.45 million new job opportunities being created each financial year, IT-BPO is India’s largest private employment sector [2,3]. This sector has not only brought in a new work culture but has also given rise to rapid changes in lifestyles, primarily making it more sedentary and associated with an increased risk of metabolic syndrome (MetS) and NCDs [4,5,6,7]. Occupational ‘burnout’ is a complex phenomenon in which the body reacts to long-term job-related stress through physical, mental and defensive coping [8] and chronic low-grade systemic inflammation caused by work stress predisposes employees to the initiation and progress of CVD and MetS [9,10,11]. In the IT industry, the complexity of IT infrastructure, poorly defined goals, resource inadequacy, extreme workload and personal inadequacy and a high requirement for accuracy have been identified as the main stressors. As such, the software industry is considered a ‘high stress’ occupation [12]. A large proportion of the workforce, specifically the younger age group (26–35 years), is exposed to these risk factors for an extended period of time before the onset of pathological NCDs [1,5]. The prevalence of MetS [12,13,14,15,16,17], along with its lifestyle risk factors [18,19,20], has been reported to be high among sedentary employees worldwide. A survey of 20,000 employees selected from different industries in India revealed that 50% were overweight, while one-third experienced central obesity, and diabetes and hypertension were prevalent in 10% and 27% of the participants, respectively [21]. However, there is no specific data on employees in the IT industry who constitute a significant portion of the working-age population and possess all lifestyle risk factors. As the prevalence of each component of MetS increases, the healthcare cost of these employees increases concomitantly [17]. Economists are of the opinion that the country’s opportunity of obtaining the demographic dividend may be jeopardised by the rising rate of NCDs among this younger working-age population. The primary reason for slowing GDP growth in India is its economic burden of NCDs, which is 5–10% of its GDP [22]. The prevention and control of metabolic risk factors are important measures to reduce the NCD burden on this economically productive population. Workplace Wellness Programmes (WWPs) generating health awareness among employees, along with the modification of the workplace environment, have been recognised by the World Health Organization as one of the most cost-effective measures of promoting the health profile of the workforce, reducing healthcare costs and ensuring enhanced productivity by reducing sick leaves and increasing ‘presenteeism’ [23]. The analytical model of WWP proposed by Genin et al. (2019) identified that in addition to occupational (environmental) factors such as the workplace environment, employer’s engagement and policies, the employee’s individual health profile, knowledge, attitude and practices play an important role in the employees’ engagement, utilisation and adhesion to their onsite WWP [24]. The assessment of health risks can form the basis of identifying the needs of employees and planning customised interventions for a WWP [25]. This report is part of a larger study that aimed to develop a flexible, multi-component, strategic model of WWP in accordance with the preventive and promotive approach to the healthcare of the National Health Policy (NHP) of India. The NHP aims to promote the lifestyle and nutrition status of employees [26], while worksite (environmental) factors such as the Food and Physical Activity Environment (FPAE) of the workplace, policies and perception of the managers about WWP were evaluated via ground truthing and Focus Group Discussions (FGDs) [27]. This part of this study aimed to assess employees’ health and nutritional status, lifestyle practices and biological markers of stress. Not only do these factors determine their risk of developing NCDs, but their individual perceptions and practices also impact health-related behaviour changes and adherence to the provided WWP. The specific objectives of this actual study, of which the current research is a part of, were to evaluate the health and nutrition status of IT employees, to estimate the prevalence of MetS among them, to assess their Knowledge Attitude and Practices (KAP) pertaining to health, food and physical activity (PA) and to evaluate the association of MetS biomarkers with perceived stress level, diet and PA practices.

## 2. Materials and Methods

### 2.1. Study Design

This research paper was part of a larger mixed-method intervention study aimed at developing, implementing and evaluating a model of WWP. In this part of the study, a cross-sectional design was utilised to evaluate the diet, PA and lifestyle-related modifiable risk factors of NCDs among IT employees to assess their nutrition status and to estimate the prevalence of MetS among them.

### 2.2. Selection of the Study Sites and Participants Recruitment

The study sites were purposively selected to include IT firms of varied operational sizes (large, medium, small) located in the south Indian City of Hyderabad, which is India’s premier information technology and IT-enabled service hub with close to 300 listed IT firms of different sizes [28]. A total of 8 organisations of varied operational sizes and no ongoing WWPs were approached, of which three IT firms of different sizes (large (>1000 employees) denoted as Worksite A, medium (>500 employees) denoted as Worksite B and small (>250 employees) denoted as worksite C in this study) consented to take part in this study. After discussion with the organisation management, promotional campaigns were conducted in all the study sites to introduce the proposed WWP to the employees. All employees of these organizations who declared themselves to be healthy and voluntarily agreed to take part were recruited in this study between June 2018 and July 2019. This method of voluntary recruitment is used in WWP studies according to workplace policy to not disrupt working schedules of employees. Employees with known chronic illness, those in transferrable job roles or planning a job switch within 1 year or those not consenting to take part in the WWP were excluded.

### 2.3. Data Collection

**Health and nutrition status of the employees:** The employees’ anthropometric, clinical and biochemical parameters were assessed. A total of 183 employees (58 employees of Worksite A, 67 employees of Worksite B and 58 employees of Worksite C) attended the physical examination, whereas *n* = 154 of them (36 employees from Worksite A, 67 employees from Worksite B and 51 employees from Worksite C) had consented for the blood tests.

#### 2.3.1. Anthropometric Measurements

Height (SECA height rod-Seca 213, Hamburg, Germany), bodyweight (digital SECA weighing scale-Seca Clara 803, Hamburg, Germany), waist and hip circumferences (flexible measuring tape) were measured. Body Mass Index (BMI) was calculated as weight in kilogram/height in square meters and categorised as per the World Health Organization (WHO) BMI cut-off points for the Asian population (BMI (kg/m^2^) of <18.5-underweight, 18.5–22.99-normal, 23–27.49-overweight and ≤27.5-obese [29]. For waist-circumference (cm), <90 for males and <80 for females were considered normal.

#### 2.3.2. Clinical Assessment

Blood pressure of each subject was measured three times at 5 min intervals in a sitting position using an Omron digital Blood Pressure Monitor (HEM-7112, Omron Healthcare Co., Ltd., Kyoto, Japan). The average of three readings was considered. As per the JNC-7 guidelines, a blood pressure (mmHg) of <120 and <80 was normal, 120–139 or 80–89 was considered pre-hypertension and a BP of >140 or ≥90 was considered as hypertension.

#### 2.3.3. Biochemical Assessment

A total of 6 mL fasting venous blood samples were collected from each employee by trained phlebotomists under the supervision of one/two of the investigators (4 mL in heparin tubes and 2 mL in EDTA tubes) and immediately moved to the laboratory on ice. Glycosylated haemoglobin (HbA1c) was estimated in the whole blood (Abott-Afinion AS100 analyser (Oslo, Norway) with the help of Abott AfinionTM HbA1c test cartridges) (HbA1c (%) Cut offs: <6-Normal, 6–6.4 Pre-Diabetes and ≥6.5 was considered diabetes-ADA, 2013). The plasma was separated, aliquoted and used for biochemical assessments, including fasting blood glucose (FBG) mg/dL (Accu-Chek^®^ Active, Mannheim, Germany) (cut off: <100-Normal, 101–126 pre-diabetes). The lipid profile (total cholesterol (TC), Low-Density Lipoprotein Cholesterol (LDL-C), High-Density Lipoprotein Cholesterol (HDL-C) and triglycerides (TG) was analysed from the plasma sample using Alere Cholestech LDX^®^ Analyzer using commercially available Alere Cholestech LDX Total Cholesterol Cassettes (Alere, San Diego, CA, USA). The cut offs used were TC (mg/dL) <200, HDL-C (mg/dL) males ≥ 40 and females ≥ 50, LDL-C (mg/dL) <130, TG < 150 (NCEP-2002).

#### 2.3.4. Assessment of Selected Biomarker Levels

The biomarkers selected for assessment and association for perceived stress level and also diet and PA levels of the study participants included MDA, Homocysteine, Interleulin-6 (IL-6) and Interleukin-4 (IL-4). This is because the reactive free radicals produced due to chronic stress react with proteins, lipids, carbohydrates and nucleic acid in the cells. In particular, the unsaturated fatty acids in the cell membrane are susceptible to oxygen radicals and undergo peroxidation. Malondialdehyde (MDA) is one of the end products of lipid peroxidation that is linked with increased cardiovascular risk, metabolic syndrome [30,31]. Increased levels of pro-inflammatory markers, IL-6 and IL-4, can accelerate atherosclerotic process and has been associated with burnout syndrome [30]. Homocysteine (Hcy) is a non-protein-forming amino acid, the concentration of which is supposedly increased during systemic inflammation and accelerates atherosclerosis by increasing lipoprotein oxidation, oxidative stress, synthesis of several pro-inflammatory cytokines, collagen synthesis, and smooth muscle cell proliferation [32]. Since these prothrombotic, proinflammatory markers are important indicators of MetS and increase the risk of CVD or diabetes, in this study, the plasma level of oxidative stress markers, MDA, inflammatory markers IL-6, IL-4 and Hcy have been estimated to assess the extent of inflammation in IT employees and associate these biomarkers with their self-perceived stress levels. MDA was estimated from plasma sample as TBARS (thiobarbituric acid-reacting substance) [33]. The concentrations of IL-4 and IL-6 in plasma were estimated via Enzyme-Linked Immunoassay (ELISA) technique using commercial kits (Human IL-4 ELISA-ImmunoTag, Geno Technology Inc., St. Louis, MO, USA. Catalogue no. ITE020199 and Human IL-6 ELISA-ImmunoTag, Geno Technology Inc., USA. Catalogue no. ITE020201). These were sandwich enzyme immunoassay kits coated with specific antibodies. The Hcy levels in the plasma samples were measured via High-Performance Liquid Chromatography (HPLC) using fluorescent detector.

**Prevalence of Metabolic Syndrome (MetS):** Although different criteria have been used in different studies to define MetS and estimate the prevalence of its individual components, in this study, we used the Harmonized Criteria (2009) [34], which is accepted worldwide to define MetS. Accordingly, the presence of three or more of the following: Waist Circumference (WC) of ≥90 cm in men, ≥80 cm in women, Triglycerides (TG) ≥ 150 mg/dL, High-Density Lipoprotein (HDL-C) < 40 mg/dL in males and <50 cm in females, Blood Pressure (BP) of ≥130/85 mmHg and FBG level of ≥100 mg/dL, with or without the presence of prothrombic and proinflammatory markers was defined as MetS [34]. Furthermore, the selected biomarkers assessed were associated with the components of MetS. The MetS components and the lifestyle health-risk factors collected with the HFL questionnaire were tested for association.

**Health, food, and physical activity related KAP:** A health, lifestyle and food questionnaire (HLFQ) questionnaire was developed by pooling and modifying the constructs from different validated questionnaires [27]. After content validation and pre-testing, the HFLQ was used to evaluate the health-, food- and PA-related KAP of the employees. The questionnaire was utilised to collect data on the employee’s socio-demographic status, general health status, dietary practices, lifestyle and PA practices, self-perceived stress and sleeping pattern. The questionnaire was closed ended with multiple choice questions or ‘Yes’ or ‘No’ answers. It was administered in an interview mode and took approximately 15 min for completion by each participant. The KAP information was collected from a total of *n =* 166 employees of the 3 worksites (39 employees of Worksite A, 67 employees of Worksite B and 60 employees of Worksite C).

**The operational definitions of the risk factors:** Dietary risk factors include skipping at least one main meal of the day (breakfast, lunch or dinner), an intake of fewer than 5 servings (400 g) of fruits and vegetables per day (WHO), addition of excess salt to food at table, frequent eating out (more than once/week).

Physical activity status: <150 min of moderate intentional PA in a week were categorised as ‘physically inactive’ (WHO).

Sedentary status or total sitting time was defined as the sum of time spent sitting during travelling, work, watching TV or using laptop and leisure.

Smoking: Those who reported smoking no cigarette at all were considered as ‘nonsmoker’, those reporting smoking at least 1 cigarette daily were categorised as ‘regular smoker’, and those reporting anything lesser than that were considered as ‘occasional smoker’.

Alcohol: In terms of alcohol consumption, those reporting no alcohol consumption were categorised as ‘non-drinkers’, people reporting consumption of alcohol at least once a week were categorised as ‘regular-drinkers’, and those consuming lesser than that were categorised as ‘occasional drinkers.

Stress level: Self-perceived stress was considered as the total score obtained by the employees on the Perceived Stress Scale (PSS), which scored them on the basis of how often they felt or thought a certain way in different situations during the last month. All reported stress scores were divided into tertiles to determine ‘low’, ‘medium’ and ‘high’ stress scores for this population.

### 2.4. Statistical Analysis

All data were processed and analysed using SPSS (Version 19.0. SPSS Inc., Chicago, IL, USA) and R Studio and R version 3.4.3 (R Development Core Team, Vienna, Austria). Since most of the data were skewed, the anthropometric, biochemical data and biomarker levels have been reported as median values, 25th percentile (P_25_) and 75th percentile (P_75_). The median values of these parameters of the MetS and non-MetS groups were compared by applying Mann–Whitney U test. The data from the questionnaire were coded and analysed using descriptive statistics. To find the correlation between different variables Spearman’s Rank Correlation was performed. Chi-square test was applied to associate age with lifestyle risk factors.

## 3. Results

During the voluntary enrolment stage, a total of 359 employees (219 employees in Worksite A, 78 employees in Worksite B and 62 employees in Worksite C) had voluntarily enrolled for the programme. However, at different stages of recruitment, there was attrition. About 66% of the enrolled participants (*n* = 359) were aged between 21 and 31 years with a mean age of 29.85 ± 6.33 years. Of these, 183 employees were assessed for anthropometry, and 154 of them were assessed for biochemical parameters.

### 3.1. Health and Nutrition Status of the Employees

**Figure 1:**
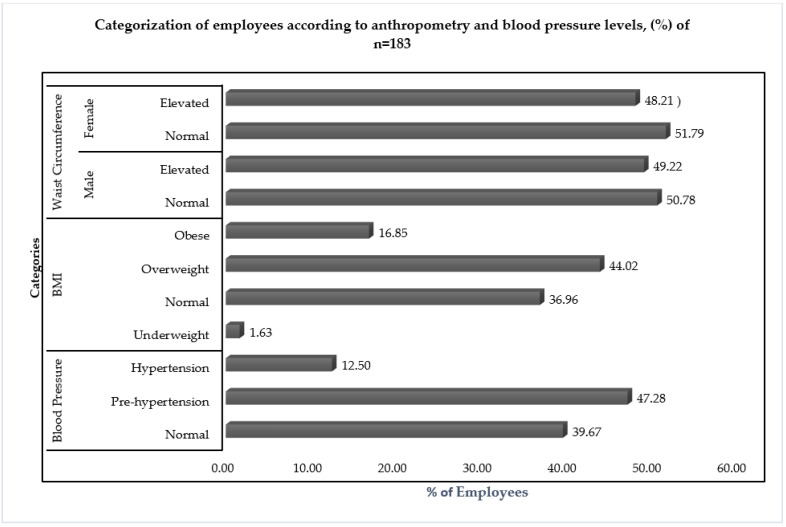
Categorisation of the participating employees on the basis of BMI, Waist Circumference and Blood Pressure levels.

Out of the 359 employees of the three IT worksites who had voluntarily enrolled for participation, only 50.97%, *n =* 183 employees (58 from Worksite A, 67 from Worksite B and 58 employees from Worksite C), had participated in the anthropometric assessment. Out of these participants, 56 (30.6%) were females. The median (P_25_–P_75_) age of these employees was 30 (26–35) years. The median (P_25_–P_75_) height was 168 (160–174) cm, and the median (P_25_–P_75_) weight was 68 (60–75.5) Kg. The median (P_25_–P_75_) values of systolic and diastolic BP were 118 (110–125) mmHg and 80 (70–84) mm Hg, respectively. The categorisation of employees as per the cut offs of BMI, Waist Circumference and BP levels is given in Figure 1.

For the biochemical assessments, 154 employees had agreed to give their blood samples (36 from Worksite A, 67 from Worksite B and 51 from Worksite C). The FBG levels revealed that 22 (14.28%) employees were pre-diabetic, and 8 (5.19%) were diabetic. Considering both FBS and HbA1c levels 12 (7.79%) employees were confirmed as pre-diabetics, and 6 (3.89%) employees were found to be diabetics. Assessment of the lipid profile showed an HDL-C level lower than recommended values in 100 (64.93%) employees. Hypertriglyceridemia was prevalent among 37.01%. The LDL-C levels were found to be high in 29.4% of employees. The median values of the biochemical parameters are given in Table 1.

### 3.2. Prevalence of Metabolic Risk Factors and Metabolic Syndrome

**Figure 2:**
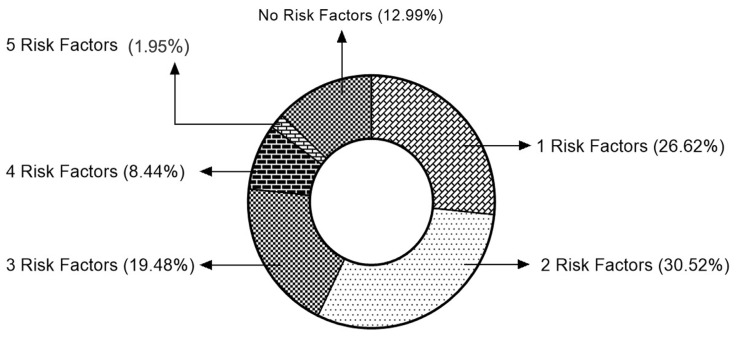
Clustering of metabolic risk factors among the participating employees.

A low HDL-C cholesterol level was the most frequently present metabolic risk factor, present in 89 (57.79%) employees, followed by a high WC in 84 (54.54%) individuals. The percentage of employees possessing each of the five metabolic syndrome risk factors is given in Table 2. In total, 20 out of 154 employees (12.98%) had none of the metabolic risk factors, while 3 (1.94%) employees had all five risk factors. Almost 46 (29.87%) of the study population were considered to have metabolic syndrome since they had ≥3 metabolic risk factors. The clustering of risk factors is given in Figure 2. The total number of employees was categorised into two groups; all employees with a MetS risk score of 0–2 were classified as No Metabolic Syndrome (No MetS), and those with a MetS risk score of 3–5 were classified as Metabolic Syndrome (MetS) group. The biomarker levels were compared between the groups.

Upon comparing the biomarker levels of the employees with No Metabolic Syndrome (No MetS) (MetS risk score of 0–2) with that of those with a MetS risk score of 3–5, the level of MDA, the end product of lipid peroxidation, pro-inflammatory markers, IL6 and IL-4 and Hcy, oxidative stress markers were found to be significantly higher among the employees who had metabolic syndrome (Table 3). The employees with MetS were also significantly older than the employees with no metabolic risk.

### 3.3. KAP of the Employees on Health, Food and Lifestyle

After removing the missing or incomplete questionnaires, a total of 166 completed questionnaires (39 from Worksite A, 67 from Worksite B and 60 employees from Worksite C) were included in the analysis. The response rate was 46.23%.

The majority of the respondents were males with a median age of 30 years. Most employees were married, and 36% had an annual income of INR > 1 million (USD 12,100). The perceived weight status of the employees when compared with actual BMI was found to be correctly perceived by 71 (42.7%) employees. The actual BMI status was higher than the perceived status in 74 (45.1%) employees and lower in 22 (13.41%) employees. About 73 employees perceived that they had no risk of developing NCDs. Although 159 (95.7%) acknowledged that maintaining an ideal body weight is essential, the most frequently (63%) mentioned reason for maintaining it was ‘to look good’, and 77.1% of participants reported to take an effort to ‘maintain their body weight’ in the form of intentional PA or by restricting food consumptions or following any specific type of diet. ‘I do not have time’ was the most frequently (37%) mentioned reason for not taking any effort. Although 79 (47.59%) employees reported being involved in intentional PA, only 38 (22%) employees reached the recommended PA duration (≥150 min of intentional PA in a week). The most frequent reason for not being involved in intentional PA was ‘I do not have time’ (89%, 78 out of 87 employees not involved in PA). The mean sitting time of the employees on a weekday was 853.12 ± 88.2 min. About 88.5% of employees reported sitting for ≥8 h a day. The prevalence of these behavioural risk factors associated with the risk of developing NCDs is given in Table 4.

Upon comparing the prevalence of lifestyle risk factors according to the age groups, it was found that even though the prevalence of MetS was significantly lower among the employees belonging to the age group of >30 years, certain lifestyle risk factors, such as skipping meals (*p* < 0.05) and frequent eating out, were significantly higher among the younger age group. Not being involved in intentional PA and higher stress level was more prevalent in the >30 years group (Table 5).

The metabolic risk score was negatively correlated with total time spent on intentional PA (rs = −0.367*) and positively correlated with total sitting time (rs = 0.287*) [*Correlation is significant at the 0.01 level (2-tailed), Spearman’s rho].

### 3.4. Association of Biomarkers with Perceived Stress Score, Diet and PA

**Figure 3:**
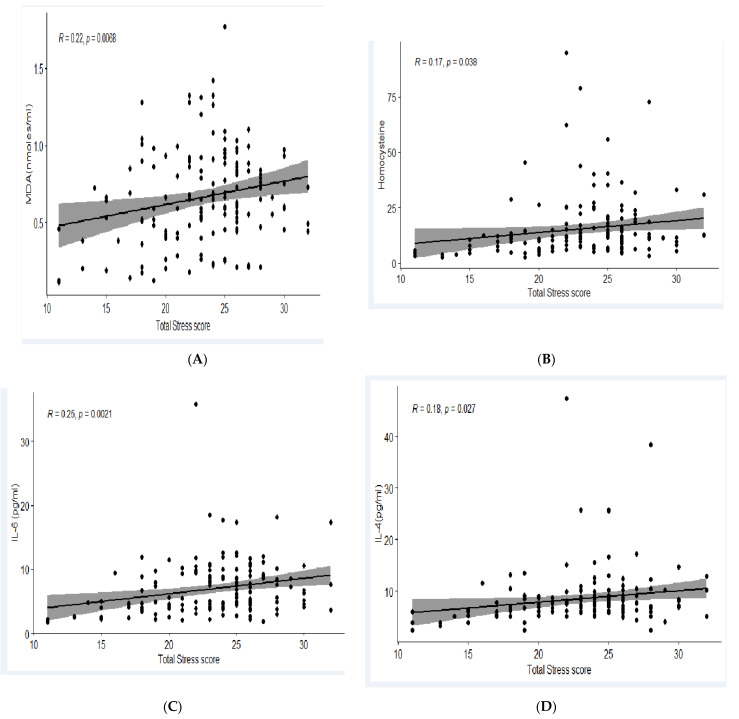
Association of specific biomarker levels with perceived stress score of the participating employees. (**A**) Association of plasma concentration of Malondialdehyde (MDA) with a total perceived stress score, (**B**) Association of plasma concentration of Homocysteine (Hcy) with a total perceived stress score, (**C**) Association of plasma concentration of Interleukin-6 (IL-6) with a total perceived stress score, (**D**) Association of plasma concentration of Interleukin-4 (IL-4) with a total perceived stress score.

The median perceived stress score (P_25_–P_75_) of the employees was 24. The total perceived stress levels were classified into tertiles: low perceived stress as <21, moderate perceived stress as 22 to 25 and high perceived stress as ≥26. The proportion of employees with high perceived stress belonged to the age group of >30 years (*p* > 0.000). The stress score was higher among those with MetS. In this study, the level of biomarkers such as MDA (R = 0.22, *p* = 0.006), HCY (R = 0.17, *p* = 0.038), IL-6 (R = 0.25, *p* = 0.002) and IL-4 (R = 0.18, *p* = 0.02) were positively and significantly associated with the perceived stress score. The elevated level of these biomarkers with increased levels of perceived stress indicates a higher risk of MetS and cardiovascular risk among the employees. The association of the perceived stress score with each biomarker is given in Figure 3.

**Figure 4:**
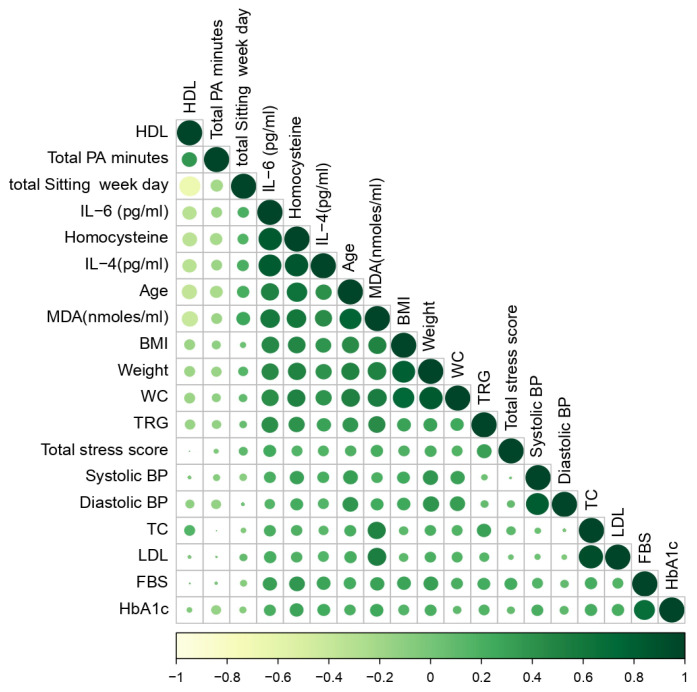
Correlation among the anthropometric, biochemical parameters and biomarkers with intentional physical activity, total sedentary time and perceived stress level. HDL-C: High-Density Lipoprotein, Total PA minutes: total time spent in a week in performing intentional physical activity, Total Sitting Weekday: total time spent in sitting while travelling, at work, watching TV or using laptop and leisure on a weekday, IL-6: interleukin 6, IL-4: interleukin 4, MDA: malondialdehyde, BMI: Body Mass Index, WC: Waist Circumference, TRG: triglycerides, BP: Blood Pressure, TC: Total Cholesterol, LDL-C: Low-Density Lipoprotein, FBS: Fasting Blood Sugar, HbA1c: glycocylated haemoglobin.

The correlogram (Figure 4) displays the correlation between each anthropometric, biochemical and biomarker with PA statuses and perceived stress levels. While an inclination towards the right side (dark green colour) represents a stronger positive correlation, an inclination towards the left represents a negative correlation.

## 4. Discussion

This study aimed to examine the prevalence of MetS risk factors among the employees of the IT industry in India. To our knowledge, this is the first study from India that associated the lifestyle, stress and dietary risk factors responsible for the development of MetS with oxidative stress biomarkers among this population. The estimation of the prevalence of MetS and the factors affecting these can help to design interventions to improve healthy behaviours among the employees. The prevalence rate of MetS among the apparently healthy sedentary workforce in different countries has been reported to range between 6.1 and as high as 58%, with a mean of 21.27% among the Asian working population [17]. In this study, a low level of HDL-C was the most prevalent component of MetS among IT employees. Different studies examining the prevalence of MetS in groups of the apparently healthy sedentary workforce globally have also reported low HDL-C as the most commonly occurring component of MetS [35,36,37].

In this current study, the average sitting time on a regular working day for the participants was found to be more than 8 h, and involvement in intentional PA was also found to be very low. This highlights the importance of including PA interventions and reducing sitting time among these employees. The studies that have included modifications to the workplace by installing facilities like standing desks and walk breaks have contributed to the reduction in sitting time [38]. In our formative study published elsewhere, it was noted that although large and medium-sized workplaces sometimes provided facilities for physical activity at the workplaces, its utilisation by the employees was poor [26]. As a part of the larger intervention in our main study, strategies were planned to make employees more physically active at workplaces [27].

Lower consumption of fruits and vegetables, frequent consumption of high-fat sugar and salt foods, skipping meals and multiple cups of sweetened tea/coffee and sugar-sweetened beverages from vending machines at the workplace have been reported in this current study. Previous studies also reported that workplace food environments with a lower availability, accessibility and promotion of fruits and vegetables had higher sales of calorie-dense snacks/meals/beverages [26]. Regarding an optimistic bias on the part of the employees that ‘they are unlikely to be at risk of developing NCDs’, only taste-driven and not health-conscious food choices were also reported among this population earlier [26]. This clearly indicates the need for modification to the workplace food environment along with intensified nutrition education and communication activities involving different modes of communication to address the individual, interpersonal and organisational factors that influence food choices [27].

In our study, the perceived stress scores were found to be higher among the senior employees, mostly at the managerial level, than the junior employees, and it was also positively correlated with the oxidative stress biomarkers (MDA, IL6, IL-4, Homocysteine). Although a study conducted on IT professionals in the north Indian state of Uttar Pradesh in 2012 reported that although the levels of overall stress were similar among the junior and senior employees, higher levels of stress were observed among the employees in their initial working years, particularly owing to job security and work overload [39]. However, these have not been associated with biomarker levels in any of these studies. This might also be because the junior employees are in a phase of familial transition and are also concerned about promotion to senior designation at their jobs and find it stressful to cope with changing expectations at work and family together. However, another study conducted among IT professionals in the south Indian city of Chennai reported higher stress levels among the more experienced and older professionals [40]. This might be because of increased responsibility [41]. Usually, the new employees are introduced to the profession with proper training in a phased manner, which helps them to become accustomed to the stress of the profession gradually. Hence, the higher levels of stress observed in our study among the senior employees compared to their junior counterparts may be because they have higher responsibility and were not introduced into the profession in a similar gradual way. The different causes of occupational stress identified in different studies include perceived job insecurity, long periods of sitting, repetitive work, lack of autonomy or resources, working late or overtime and organisational environment [42,43]. In our study, although workload and meeting deadlines were most frequently pointed out as the major stress factors at work, lack of team support or job dissatisfaction were not mentioned by anyone. It might be because the industry provides satisfying infrastructure and motivating factors to its employees. Some of the studies suggest that exposure to job stress over 14 years increases the risk of developing MetS significantly [44,45], and overall, IT job roles have been considered to possess higher stress levels than other professions. Hence, stress management both at the individual and organisational levels through relaxation techniques, meditation, exercise and changing physical and environmental characteristics of jobs can be adopted as a part of WWPs as an NCD preventive strategy.

One of the limitations of this current study is that the sample size was small and may not be representative of the IT workforce in India. However, the overall prevalence of MetS among almost a third of those studied was higher than that of the average Asian working population, which, indeed, is a cause of growing concern. Yet another limitation of this current study is its limited geographic coverage as it was primarily based in one technology hub of India, though there are several other emerging hubs. However, this study is in concurrence with several other studies that indicate that overweight and obesity among the economically productive young population of the country is one of the highest in South East Asia, creating a looming danger to the country’s economy. The situation might be similar in other parts of the world with the booming growth of the Information Technology sector. One method of improving the health of this large section of the productive workforce is through workplace wellness programmes with a conscious inclusion of nutrition and physical activity promotion programmes. The health risks of the employees can be addressed with changes in workplace culture, organisational policies and, most importantly, by changing the attitude of the employees and employers. Since there is no established workplace wellness model and each organisation is unique, before planning any workplace wellness strategies, situational analyses should be conducted, and our study can serve as a model.

## 5. Conclusions

The biochemical parameters, lifestyle risk factors and components of MetS among the study participants indicate a high risk of developing chronic diseases among the employees of the IT industry in India. Although, in this study, the employees with MetS were significantly older than those without MetS, the lifestyles and associated risk factors for NCDs were prevalent even among the younger employees. This shows the importance of modification of lifestyle and work culture of the employees.

## Figures and Tables

**Table 1:** Median (P_25_–P_75_) values of the biochemical parameters of all participating employees (*n =* 154).

Biochemical Parameters	Number of Participants (*n* = 154) and Median (P_25_–P_75_) Values
Fasting Blood Glucose (FBG) mg/dL	88 (82–93.5)
Glycosylated Haemoglobin (HbA1c %)	5.6 (5.3–5.8)
Total Cholesterol (TC) mg/dL	175.6 (150.4–212)
High-Density Lipoprotein (HDL-C) mg/dL	40 (32–48)
Low-Density Lipoprotein (LDL-C) mg/dL	105 (87–142.5)
Triglycerides (TG) mg/dL	128.5 (104.5–157)

**Table 2:** Prevalence of each component of Metabolic Syndrome across the study population.

Metabolic Risk Factor	Percentage of Study Population (*n* = 154)
	*n* (%)
Elevated Blood pressure (≥130/85 mmHg)	32 (20.77)
Low High-density lipoprotein (<40 mg/dL-Males, <50 mg/dL Females)	89 (57.79)
High Triglycerides (≥150 mg/dL)	57 (37.01)
Elevated Fasting Blood Sugar (≥100 mg/dL)	30 (19.48)
Elevated Waist Circumference (Males-≥90 cm, Females ≥80 cm)	84 (54.54)

**Table 3:** Comparison of biomarker levels between the employees with No MetS and those with MetS.

Parameters	No MetS (Score 0–2)	MetS (Score 3–5)	*p* Value
Age (Years)	28 (21–40)	38.5 (22–50)	0.000 *
Homocysteine (µmole/L)	9.80 (2.66–23.95)	25.37 (5.7–94.94)	0.001 *
MDA (nmoles/mL)	0.55 (0.12–1.09)	0.92 (0.6–1.77)	0.003 *
IL-6 (pg/mL)	4.60 (1.67–12.04)	9.5 (4.63–35.86)	0.017 *
IL-4 (pg/mL)	6.56 (2.34–13.2)	9.4 (6.18–47.45)	0.000 *

* Mann–Whitney U test.

**Table 4:** Prevalence of behavioural risk factors associated with developing NCDs among the study participants.

Behavioural Risk Factors	Indicators	Prevalence (*n*, %)
Dietary Risk Factors	Skipping at least 1 meal every day	50 (30.12%)
	Consuming >400 g of fruits or vegetables every day	64 (38.55%)
	Adding extra salt to food on table	51 (30.7%)
	Eating out frequency > once every week	111 (66.8%)
Activity Status	Not involved in intentional physical activity	87 (52.4%)
	Total intentional physical activity time <150 min/week	128 (77.10)
	Total sitting time >8 h/day	147 (88.55%)
Smoking habits	Regular smoking	37 (22.2%)
Alcohol consumption	Regular consumption of alcohol	35 (0.21%)
Stress Level	High self-perceived stress level (stress score ≥ 26)	56 (33.73%)

**Table 5:** Comparison of prevalence of risk factors between employees of different age groups.

Risk Factors	<30 Years (n, %)	≥30 Years (n, %)	X^2^	Significance
Skipping meals	39 (50.6)	8 (10.4)	29.42	0.000 *
<400 g of fruits & veggies	46 (59.7)	55 (71.4)	2.33	0.127
Adding extra salt	19 (24.7	26 (33.8)	1.53	0.215
Frequent eating out	72 (93.5)	51 (66.3)	23.93	0.000 *
>8 h sitting time/day	68 (83.3)	71 (92.2)	.665	0.415
No involvement in intentional physical activity	31 (40.3)	52 (67.5)	11.52	0.001 *
<150 min Physical Activity/week	56 (72.7)	68 (88.3)	5.96	0.015
Smoking	34 (44.2)	42 (54.5)	3.85	0.197
Alcoholism	45 (58.5)	45 (58.5)	3.87	0.144
High perceived Stress	47 (61.1)	63 (81.8)	9.48	0.000 *
Metabolic syndrome	2 (2.6)	44 (28.57)	5.468	0.000 *

* Chi-square test *p* < 0.005.

## Data Availability

Data cannot be shared at this time due to ethical reasons and privacy requested by the participating companies.
